# From SNPs to pathways: Biological interpretation of type 2 diabetes (T2DM) genome wide association study (GWAS) results

**DOI:** 10.1371/journal.pone.0193515

**Published:** 2018-04-04

**Authors:** Elisa Cirillo, Martina Kutmon, Manuel Gonzalez Hernandez, Tom Hooimeijer, Michiel E. Adriaens, Lars M. T. Eijssen, Laurence D. Parnell, Susan L. Coort, Chris T. Evelo

**Affiliations:** 1 Department of Bioinformatics – BiGCaT, NUTRIM School of Nutrition and Translational Research in Metabolism, Maastricht University, Maastricht, the Netherlands; 2 Maastricht Centre for Systems Biology (MaCSBio), Maastricht University, Maastricht, the Netherlands; 3 Agricultural Research Service, USDA, Jean Mayer-USDA Human Nutrition Research Center on Aging at Tufts University, Boston, MA, United States of America; Huazhong Normal University, CHINA

## Abstract

Genome-wide association studies (GWAS) have become a common method for discovery of gene-disease relationships, in particular for complex diseases like Type 2 Diabetes Mellitus (T2DM). The experience with GWAS analysis has revealed that the genetic risk for complex diseases involves cumulative, small effects of many genes and only some genes with a moderate effect. In order to explore the complexity of the relationships between T2DM genes and their potential function at the process level as effected by polymorphism effects, a secondary analysis of a GWAS meta-analysis is presented. Network analysis, pathway information and integration of different types of biological information such as eQTLs and gene-environment interactions are used to elucidate the biological context of the genetic variants and to perform an analysis based on data visualization. We selected a T2DM dataset from a GWAS meta-analysis, and extracted 1,971 SNPs associated with T2DM. We mapped 580 SNPs to 360 genes, and then selected 460 pathways containing these genes from the curated collection of WikiPathways. We then created and analyzed SNP-gene and SNP-gene-pathway network modules in Cytoscape. A focus on genes with robust connections to pathways permitted identification of many T2DM pertinent pathways. However, numerous genes lack literature evidence of association with T2DM. We also speculate on the genes in specific network structures obtained in the SNP-gene network, such as gene-SNP-gene modules. Finally, we selected genes relevant to T2DM from our SNP-gene-pathway network, using different sources that reveal gene-environment interactions and eQTLs. We confirmed functions relevant to T2DM for many genes and have identified some—*LPL* and *APOB*—that require further validation to clarify their involvement in T2DM.

## Introduction

### GWAS and pathway analysis

Since 2005 analysis of genetic variations in complex diseases has been conducted with genome-wide association studies (GWAS) [1]. Such an analysis consists of genotyping the genomic DNA of individuals divided into case and control groups according to a specific trait or phenotype. A genome scan is performed using a set of genetic variation probes of at least 100,000 single nucleotide polymorphisms (SNPs) to a million or more, and more recently genomic sequencing-based approaches to detect SNPs have been added [2]. Thereafter, computational methods are applied to SNPs related to the investigated phenotype, resulting in a list of SNPs significantly associated with the phenotype. Despite the limitations of such studies [3], GWAS is still a valuable method that provides insights to delineate the molecular scenario of complex diseases like type 2 diabetes mellitus (T2DM) and to support risk prediction [4].

Nevertheless, it remains challenging to perform secondary analysis on GWAS results with the aim of obtaining a detailed biological understanding of the SNPs function and role in a disease [5]. Pathway analysis is an example of secondary analysis that has been applied to GWAS since 2007 [6], where the SNPs are contextualized in biological processes through the genes to which they are assigned [7]. Garcia-Campos et al. (2015) and Kai et al. (2015) [8, 9] published reviews describing how to use different pathway analysis methodologies, which are applicable to GWAS data. Three basic steps are performed in these pathway analysis methods: (1) gene set are chosen by the user for instance from Gene Ontology annotations [10], KEGG or WikiPathways pathways [11], (2) genetic variants are mapped onto the genes, and (3) gene set statistics are performed. There are two different approaches for the gene set statistics: in a one-step approach, gene set p-values are calculated directly from genotype data, whereas in a two-step approach first single gene p-values are determined, from which the final gene set p-values are computed.

Another way to discriminate gene set statistical methods is by the difference in the hypothesis tested. The hypothesis tested is either whether the observed pathway is associated with the phenotype (often referred to as a self-contained approach or an association method), or whether the genes within a pathway are significantly enriched in comparison of other genes (referred to as competitive approach or enrichment method). In both cases the output is a list of pathways ranked by their significance based on the statistical test performed. Gene set enrichment was used to obtain interesting and germane pathway results linked to diabetes [5, 12]. However, looking only at the highest ranked pathways does not assure an accounting of all genes detected by the significant association signals, interpretation of which could be relevant to understand the phenotype. In general, it is often suggested that the output list needs to be checked manually, pathway by pathway and gene by gene. It then becomes time-consuming and error prone to account for all the possible relations that different pathways and genes present between each other.

We propose an approach based on network analysis and visualization where we display biological pathways identified by the presence of genes in which the SNPs from T2DM GWAS meta-analysis are mapped. The list of pathways is derived simply from the fact that one or more genes associated with a significant GWAS SNP signal are present in that pathway. This allows us to create a SNP-gene-pathway network that includes all the pathways where a significant SNP was found.

Furthermore, in recent years development of methods for testing hypotheses about the molecular mechanisms of a phenotype from the GWAS results, has promoted several secondary approaches besides those related to pathway-based analysis [13]. An example is expression quantitative trait loci (eQTL) analysis that can enhance the characterization of GWAS variants, in particular the non-coding ones. eQTLs are loci that contain sequence variants that are found to affect the expression of genes. They are identified by relating gene expression measurements to genotyping information in panels of individuals [14]. eQTL databases, such as GTEx portal [15], provide the opportunity to link GWAS results to the transcriptome level, in which a GWAS hit matching an eQTL for a given gene, brings up the hypothesis that the expression of this gene influences the particular phenotype. This transcript level can be analyzed in tissues relevant to the phenotype of interest. Pre-identified gene-phenotype and gene-environment interactions also form important resources because they allow us to both confirm the relation between the environmental causes that could lead to the gene network found (for instance determined by nutrition), and the relation between the gene network and the disease phenotype, in order to identify subnetworks related to more specific aspects of the phenotype (*e.g.* inflammation). CardioGxE is an important resource for such gene-environment and gene-phenotype interactions [16]. We use GTEx and CardioGxE to better understand the SNP and gene connections in the network, in relation to pathway context, environmental causes and the T2DM phenotype.

### Key pathways in type 2 diabetes mellitus

We chose to perform an analysis on T2DM data because this is a highly investigated complex disease. There are many and different types of T2DM data and biological information published in articles or stored in databases that can be re-used and integrated for secondary analysis [17]. T2DM is the inability to regulate glucose levels in the blood associated with the development of insulin resistance [18]. This insulin resistance can be systemic or tissue specific. The high glucose levels progressively stress the pancreatic beta-cells, which respond by increasing secretion of insulin. Insulin induces glucose uptake in skeletal muscle, and regulates both glucose production in the liver and the release of free fatty acids from adipose tissue. The insulin imbalance leads to complications related to those organs. Pathway analysis results of T2DM GWAS studies [12, 19] have identified molecular pathways involved in the tissues previously mentioned such as: pancreas, liver, adipose and skeletal muscle. For example, the G-protein signaling pathways are known to activate genes like mitogen-activated protein kinases (MAPK) resulting in insulin resistance, regulation of lipid metabolism, and calcium signaling that converges in AKT signaling and promotes glucose uptake in response to insulin [12]. Another example is the neural development processes found to be enriched with well-known T2DM genes like *TCF7L2* that also have a role in the WNT-signaling pathway, involved in the regulation of pancreatic development [19].

Identifying the genetic influence on the pathways implicated in T2DM pathophysiology is an influential step to determine the genetic predisposition of this complex disease, and offers targets for development of pharmacological agents. In this study we apply a novel network biology approach to identify genes and pathways relevant in T2DM based on GWAS results. To gain more insights into the possible role of identified genes in T2DM, we use several databases and literature search tools. Lastly, we identified a number of genes with known influence on T2DM phenotypes and others with potential molecular roles in T2DM, but which require further validation.

## Materials and methods

### GWAS dataset

The GWAS results described in the current study are taken from a human GWAS meta-analysis conducted by Johnson and O’Donnell in 2009 [20]. The authors used a custom computer analysis to extract and collect 56,411 significant SNP-phenotype associations, in an publicly available GWAS database, from 118 previously published GWAS studies related to different phenotypes. As stated in the paper “the database represents results from an heterogenous set of studies with varied amounts and types of data available”. The description of the included studies, and the information on how the meta-analysis was conducted (*i.e.* search strategy, study quality, heterogeneity between study variance, etc) are reported in the Method and Material section of the original paper [20]. We extracted 1,971 SNPs (in August 2016) associated with T2DM, which came from nine of the T2DM GWAS articles collected [21–29], and a total of 22,363 samples were considered. From this pool of SNPs 1,621 SNPs are from populations with European ancestry (and hence highly relevant to LD analysis performed with CEU data), 195 SNPs are from a MEX population and 155 SNPs are from American Indians. Study information for these articles related to: number of cases and controls, genotyping arrays used, phenotype descriptions, replication samples, analytic strategies, data availability, URLs, publication date and contact information are listed in additional file 2 and 3 of the dataset publication [20].

Consequently, in December 2017, an additional 757 SNPs associated with T2DM were retrieved from the GWAS Catalog (https://www.ebi.ac.uk/gwas/) and their genes and pathways were investigated. Those SNPs are not present in the Johnson and O’Donnell dataset, because they were detected in studies performed after their analysis. We report the full list of genes and pathways related to the 757 SNPs as Supplemental material in S1 Table.

### Workflow of the analysis

**Fig 1:**
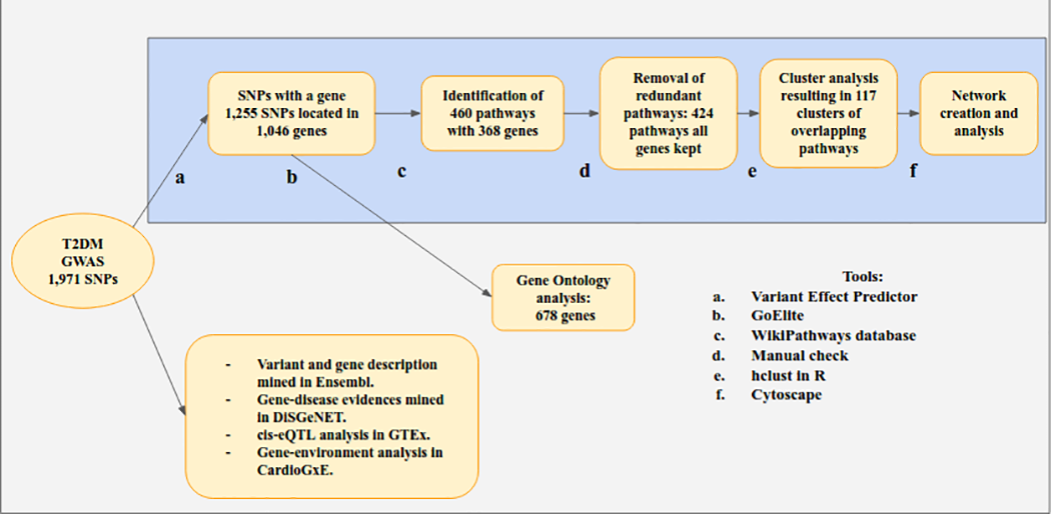
Workflow of GWAS data analysis. The data processing, online resources and tools used to perform the GWAS data analysis and visualization, as described in Materials and Methods.

Our workflow of the data analysis is presented in Fig 1 showing the different steps and tools used. We started our analysis by mapping the 1,971 T2DM-linked SNPs to the genome using the Variant Effect Prediction tool (VEP [30]). This tool gives both the chromosomal location and the known consequences of the variants in the gene sequence (as defined by Sequence Ontology [31]), transcripts, proteins, and regulatory regions (http://www.ensembl.org/info/docs/tools/vep/index.html). We obtained 716 variants located in intergenic regions and 1,255 SNPs positioned within 1 kbp up and downstream of the 5’ and 3’ UTR of 1,046 genes.

We used Ensembl BioMart to retrieve the Ensembl gene identifier and gene name for the 1,046 genes linked to the T2DM SNPs [32]. Next, we checked their pathway involvement using the human complete WikiPathways curated collection [11] (Analysis performed in October 2016, 710 pathways). We identified 368 of 1,048 genes in 460 different pathways.

The 678 genes not present in any of the pathways were annotated with Gene Ontology (GO) terms using GOElite [33] in which 672 genes were detected in the three top level Gene Ontology (GO) trees: molecular function, biological process and cellular component. The GO annotation of these 672 genes was obtained running the GOElite analysis with default parameters (Z-score cutoff for initial filtering above 1.96, 3 minimum number of genes changed (genes connected to a GO term), permutated p-value cutoff above 0.05, excluding terms with gene ID counts greater than 10000). Then, complete results list was used without taking into account the parameters chosen such as: Z score or number of genes changed. As we were trying to complement the biological pathway analysis, we then focused the investigation on the 1,503 GO terms (associated with 196 genes from the original 672) found in the biological processes tree. Furthermore, we used the pathway list obtained from WikiPathways in combination with the gene-variant relations retrieved from BioMart to create a SNP-gene-pathway network, using Cytoscape 3.3.0 [34]. This network contains nodes for all three types of entities. Pathways are included whenever they contain one or more genes that were found to be associated with one or more of the SNPs. These genes then become part of the network and are connected to all pathway nodes in which they occur. Finally, SNPs are connected to the gene(s) to which they were mapped.

**Fig 2:**
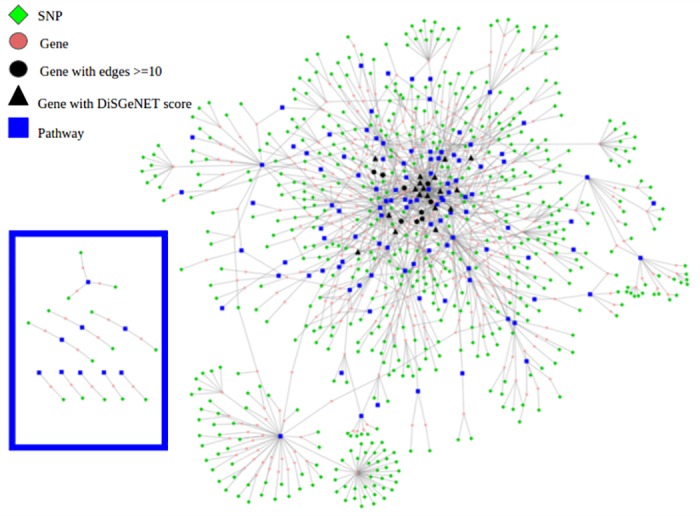
SNP-gene-pathway network. The network displays 580 SNPs (green diamonds) located in the selected region for 365 genes (circles) present in 117 pathway clusters (blue squares). Black symbols indicate genes with ten or more connections to pathway clusters, and triangles indicate genes with a positive DisGeNET score (note that these are all black). The disconnected SNP-gene-pathway subnetworks are shown on the left, framed in black.

Before building this T2DM network, we implemented procedures that allowed us to reduce the redundancy of the pathways used. The aim of this was to obtain a less crowded visualization, without losing the relevance of given networked pathways. We manually evaluated the pathway names and content and whenever two were found with similar names and overlapping content, the smallest pathway was removed. This led to 36 pathways being excluded. On the remaining 424 pathways we performed a cluster analysis in R using the “hclust” function with Euclidean distance and complete linkage clustering. We obtained 81 clusters of pathways and 36 individual pathways. We displayed the resulting 117 pathway clusters as a network in Fig 2.

### Information sources for GWAS data interpretation

We used several types of online sources and performed web-based analyses to retrieve specific biological information regarding SNPs and the genes to which they are related. We include these additional steps in the workflow Fig 1. The information retrieved from those sources enhanced the understanding of the relations between SNPs, genes and pathways.

Regarding the SNPs the following database and analyses were performed:

1) SNP descriptions were obtained from Ensembl VEP, in which we checked the variant location and the consequences of the variation on the DNA sequence (*i.e*. intronic, missense, regulatory, etc).

2) Ensembl Variation database was consulted directly to find citations related to the variants (queried by rs IDs) and to the diabetic phenotype, and the chromatin state regarding the variant location.

3) A Pairwise Linkage Disequilibrium test on the T2DM SNPs with genes was performed for the CEU and MEX SNPs, using the SNP annotation tool SNAP (http://archive.broadinstitute.org/mpg/snap/ldsearchpw.php) [35]. The number of CEU SNPs is 504, the MEX SNPs is 50 and the American Indians is 28, and in S1 Fig the SNPs with genes are colored differently according to the population in which they were detected. The parameters used were: HapMap 3 (release 2), CEU population for the 504 SNPs and MEX population for the 50 SNPs, r2 threshold 0.8, and Distance limit 500 kb. The LD test was not performed for the American Indian population because of the lack of a suitable genetic dataset.

4) The T2DM SNP list was used to query the CardioGxE database [16] to verify if any GWAS variant was previously reported to have a significant gene-environment interaction.

5) The entire set of GWAS, with data from 7,051 post-mortem samples representing 44 tissues and 449 individuals was queried at the GTEx portal [15, 36] in order to identify cis-eQTL. Particular to the GTEx data, we first queried the 716 SNPs not mapping in or near genes to retrieve eQTL data in any tissue. Then we checked for cis-QTLs for the 1,255 SNPs that map in or near genes and the genes to which they map for eQTLs in four T2DM relevant tissues: pancreas, liver, subcutaneous adipose tissue, and skeletal muscle. Finally, the results were visualized in a Venn diagram created with Venny [37].

Concerning gene information, we manually retrieved functional gene descriptions for the genes from GeneCards (http://www.genecards.org/) [38] and disease associations from DisGeNET (http://www.disgenet.org/) [39]. The evidence obtained was sorted using “Diabetes” as a keyword and the article pertinence was evaluated using both the DisGeNET score and reading the article. DisGeNET score ranks gene-disease associations according to their level of evidence calculated by an algorithm that considers the number and type of sources present in the database, and the number of publications that support the association. For some genes we also performed an additional search using PubMed and Google Scholar in which a query of “gene name AND Diabetes” was used. From the list of articles retrieved, abstracts were scanned, and only those that reported gene name and type 2 diabetes were further analyzed.

As a last step, a search of WikiPathways was used to read the details of the Diabetes related pathway diagrams (*i.e.* Description, Ontology tag, etc) and to evaluate the role of the identified genes.

## Results and discussion

### SNP analysis

The 1,971 SNPs associated with T2DM were analyzed using different tools and database information from the resources mentioned above, in order to have a detailed description of the variants, the genes and the pathways that can lead to plausible biological mechanisms regarding T2DM risk or onset or progression of this disease. 98% of T2DM SNPs were mapped to non-coding regions of which 716 SNPs (36%) were outside gene regions (called intergenic variants). The percentage of total non-coding variants in the dataset is consistent with what other GWAS studies detect [3]. The non-coding variants are described in more detail in the section that covers the eQTL analysis.

The coding SNPs in the dataset consist of: two nonsense SNPs: rs328 and rs2499953, each with high impact on the *LPL* and *MMP26* genes respectively, five missense SNPs (rs2271586, rs5215, rs2499953, rs10494217, rs13088) with moderate impact on the gene protein function according to SIFT and PolyPhen scores, and six synonymous variants with no resulting change to the encoded amino acid, but which nonetheless may alter translation rates and protein structure and function [40].

Regarding the genes with high-impact mutations, *LPL* is a lipoprotein lipase and its mutations increase ending diabetes mellitus. Matrix metalloproteinases (MMPs) are proteolytic enzymes belonging to the family of zinc-dependent endopeptidases that are risk of hyperlipidemia, a known complication in T2DM. *LPL* is a key enzyme in human lipid metabolism that facilitates the removal of triglyceride-rich lipoproteins from the bloodstream [41, 42]. For *MMP26* a risk allele was reported to be associated with higher fasting plasma glucose [43], and the *MMP26* gene is known to have a role in diabetic nephropathy occurring after a longstcapable of degrading almost all the proteinaceous components of the extracellular matrix. It is known that MMPs play a role in a number of renal diseases, such as various forms of glomerulonephritis and tubular diseases, including some of the inherited kidney diseases [44]. The fact that *MMP26* is a T2DM GWAS hit and carries a nonsense SNPs involved in T2DM complications, is sufficient to warrant further investigation of this gene and its involvement in T2DM.

Finally, identification of proxy SNPs allowed determination of redundancy within each dataset. Strong LD (r2 > 0.8) between CEU SNPs was found in 17% of the 504 variants with genes, and for the MEX SNPs strong LD was present in 24% of the 50 SNPs with genes. We also identified genes where the SNPs are in strong LD only in MEX, such as *OR51A7*. Nonetheless, pathway analysis was performed for all genes, identified in populations of European or non-European ancestry, because pathway function and disease phenotypes are highly conserved across populations.

### Analysis of the SNP-gene-pathway network

The curated human WikiPathways collection was used to retrieve pathway information on the genes related to the T2DM GWAS SNPs. In WikiPathways genes and metabolites are connected by lines that show meaningful interactions and/or chemical reactions between entities present in the pathway. From the 1,046 SNP related genes identified via Ensembl BioMart, 368 were found in a total of 460 pathways of the complete 709 pathways in the WikiPathways curated collection. In order to achieve a comprehensive picture of the biological processes related to all genes and pathways detected by the T2DM SNPs, a SNP-gene-pathway network was created, following the steps explained in the blue box of the workflow in Fig 1. The network consists of 580 SNPs located in 368 genes present in 460 pathways, which in Fig 2 are shown as 117 cluster nodes. Cluster nodes were created by merging redundant pathways that share the same genes as reported in the methods section. Furthermore, to better describe and discuss the biological connections of the network nodes, we conducted both a network topology investigation and an integration of information from other data sources. In particular, we studied the node degree distribution using the Cytoscape NetworkAnalyzer module [45] and we integrated additional information from other databases and sources, such as: GeneCards (gene description), Ensembl (gene and variants description), DisGeNET (evidences on gene-disease association), CardioGxE (gene-environment interaction), and PubMed and Google Scholar (for confirmation of gene function).

### Focus on the gene-pathway connections

For each gene node, the number of gene-pathway connections (node degree), was calculated to detect which genes have the highest number of connections to pathways. 27 genes were found to be connected to ten or more pathways even after removal of redundant pathways. These genes are located in the core of the network. The list shows either pleiotropic genes such as transcription factors (*i.e. NFBK1, CREB1*) and serine/threonine kinases (*i.e. PRKCA, CHUK, JAK2*) or typical T2DM genes (*i.e PPARG*). Furthermore, we explored the gene-disease association in T2DM using DisGeNET, and we summarized the findings in S2 Table. For 18 of the 27 core genes in the network we found evidence related to T2DM phenotype in DisGeNET. The DisGeNET score for these 18 varied from 0.001 for *JAK2, MAP3K1*, and *TGFBR1* to 0.393 for the most T2DM associated gene *PPARG*. The scores rank the gene-disease associations according to their level of evidence, range from 0 to 1, with the higher score indicating greater confidence in the gene-disease association. The genes with a positive DiSGeNET score are displayed as a black triangle in Fig 2. We explored the pathways shared by at least 2 of the 27 core genes and found 161 common pathways. These pathways were then clustered using the pathway ontology tags present on WikiPathways. The main clusters with most contributing pathways were: pathways related to immunity (*e.g.* B-cell and T-cell receptor signaling, Toll-like receptor signaling, TNF alpha, interferon type I and Interleukin 11 signaling), neuron activity (BDNF signaling and Neurotransmitter receptor binding), cell life cycle related pathways (*e.g.* apoptosis, MyD88 cascade initiated on plasma membrane and endosome), hormone signaling pathways (*e.g.* androgen and estrogen signaling), energy related pathways (leptin, insulin and AGE/RAGE signaling), heart function related pathways (*e.g.* cardiac hypertrophic response) and different types of signaling pathways (*e.g.* EGF/EGFR ErbB, MAPK signaling etc.). The variety of these pathways can be explained by the pleiotropic action of genes such as kinases or transcription factors, and it is remarkable to observe that, according to previous knowledge, many of these pathways are relevant to T2DM pathophysiology.

All 27 genes are located in the core of the large central network which consists of the nodes with the highest connections. However, nine small network structures are disconnected from the larger network, these consist of fourteen genes (see the black frame on the left side of Fig 2). The fact that most of the 368 genes are connected in a central network indicates that they function in pathways that are interlinked. Sharing genes between pathways can in fact point at different forms of relations between pathways. Pathways can be functionally related (one regulates the other, or metabolites move each) and can describe related processes in different ways (the strong relationships between cell cycle and cancer pathways, for example) and the shared genes can have real pleiotropic functionality where they play different roles in the two pathways. The latter typically happens for instance for transcription factors that can have multiple targets which can appear in different pathways.

The disconnected small network structures that are represented in the frame in Fig 2 consist of a single pathway node with the associated genes. These pathways are: 1) energy related pathways (peroxisomal lipid metabolism, bile acid metabolism, glycolysis and gluconeogenesis, TCA cycle and respiratory electron transport), 2) neuron related pathways (synaptic vesicle, GABA metabolism and dopaminergic neuron), 3) pathways related to general cellular processes (RNA transcription, RNA processing and oxidative phosphorylation, and 4) the ACE inhibitor pathway. All these processes have a clear and known involvement in T2DM pathophysiology [12, 18, 19] and there are many known connections between these processes and other parts of the larger network. Some of these pathways are separate from other similar pathways present in the larger network, because the genes known to connect them were not found to have associated SNPs. The gene-pathway isolation within the network could also be the result of incomplete knowledge representations in the pathways, where disease associated genes are not represented in each of the related pathways, not because the pathways as such are unrelated.

Our analysis helps to identify both potential mechanistic links between pathway (sub)networks and an understanding of epistasis (SNP-SNP or gene-gene interactions) [46] that is supported by SNPs mapping to different pathways. We found an example of a missing link between the unconnected pathways previously listed, for instance between the TCA cycle (WikiPathways ID: WP2766) and the glycolysis and gluconeogenesis (WP534) pathway. The two processes are clearly related but the conceptual division can be made in such a way that no genes are shared. WikiPathways in fact has a mechanism to show this: the pathway diagram can have an explicit link to another pathway diagram. However, this pathway-pathway relation is still hard to interpret in the type of network that we presented here, because we identify a connection between a gene in one pathway and an entire other pathway, and not with a specific gene (pathway 1) to gene (pathway 2) connection. We also found an example of a missing link between the unconnected processes and the processes in the large central network, regarding again the TCA cycle and glycolysis and gluconeogenesis pathways, and a pathway that describes the transport of glucose and other sugars (WP1935). The latter pathway contain the SLC family of glucose transporters, of which seven SLC genes are present in the network, but without links to the main glucose metabolism related pathways (WP2766 and WP534). These findings are useful to improve the WikiPathways collection, where we could add pathway links to all three diagrams. Clarifying the functional connections between genes in different processes that contribute to the synergistic effects found in GWAS studies, represented in our network, helps to understand the background of epistasis.

### Focus on the SNP-gene connections

Several genes have a relatively high number of SNPs associated with T2DM (*CNTN1, GRB10, PRKCA, ZNF615, SYNE1, THSD7B, NRG1, DDOST, SLC13A1, HIPK2, ATP8A1, ARHGAP26, TCF7L2, CDKAL1*). The S3 Table gives an overview of the number of related SNPs found for every gene. It should be noted that some SNPs are in high linkage disequilibrum (LD) and thus would point to the same causal variant. For example there are four SNP-gene network structures with a number of SNPs greater than ten. The genes at the centers of these networks are: *CDKAL1, ATP8A1, ARHGAP*, and *TCF7L2*, but considering LD only the gene *CDKAL1* remains connected to at least 10 SNPs independent of each other. *CDKAL1* variants have been reported to be associated with T2DM with highly significant p-values detected by different GWAS studies [5].

In the blue frame of S1 Fig 19 structures are placed, in which one or more SNPs connect with at least two genes, meaning that those SNPs are located in multiple genes according to the size of the gene regions chosen. Such genetic overlaps are well known, and they are important for the biological interpretation of the outcome from GWAS studies. In such cases the knowledge of the function of the associated genes can be used to decide which relations are more plausible [47]. For this purpose we collected gene-disease associations with T2DM and their scores from the DisGeNET database regarding the 41 genes present in the 19 structures, genes with such scores are shown as black triangles in Fig 2, and we also report the DisGeNET score that indicate strength of the gene-disease association in S4 Table. The DisGeNET analysis reveals that 13 of the 41 genes found to have SNPs associated with T2DM in the GWAS analysis already have known relationships with T2DM (31%), a value likely the result of evaluation of the same GWAS studies. However, if when considering the SNPs in S4 Table that overlap with multiple genes, SNPs associated with these 13 previously known T2DM genes are also associated with 8 other genes. In some cases such a dually connected SNP is the only association found for a specific gene, which reduces the likelihood that such a disease relationship is real.

Finally, we checked if any of the SNPs and related genes in the network presented a known gene-environment interaction in the CardioGxE database. Finding relevant gene-environment interactions in the database adds supports for their involvement in the disease mechanism. We found six SNPs (rs9939609, rs1801282, rs7903146, rs328, rs693 and rs780094) influenced by thirteen environmental factors such as: energy intake, whole-grain intake, fiber intake, carbohydrate, fat, polyunsaturated, monounsaturated and saturated fatty acid, Vitamin E and A, normal diet, Mediterranean diet and physical activity. Those SNPs are located in six genes (*FTO, PPARG, TCF7L2, LPL, APOB*, and *GCKR* respectively), most of which are well-known to be associated with T2DM and its complications [4, 18]. CardioGxE also provides a list of phenotypic traits related to these genes such as: body mass index (BMI), insulin, triglyceride, and cholesterol. The complete results of the gene-environment interactions are reported in S5 Table.

### Gene Ontology analysis of the genes without pathways

**Fig 3:**
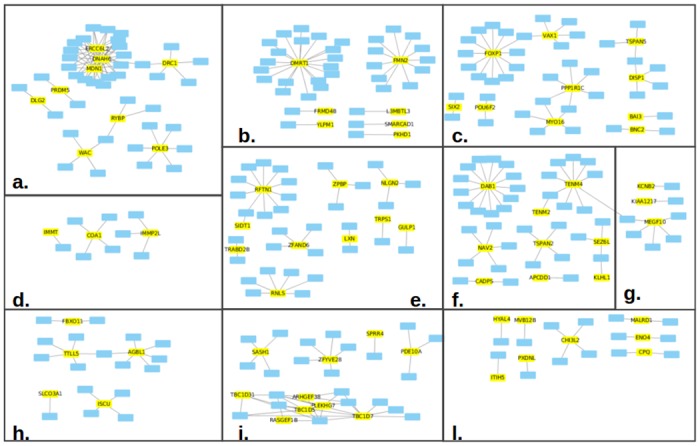
Overview of the GO biological processes exclusively linked to genes without pathways. The image represents 248 GO biological processes (blue rectangles) linked with 74 genes without pathways (yellow rectangles). The processes are grouped in ten frames according to similar functions. Each group is identified with the highest level GO term that identifies the general action of the processes. a. DNA modification (*e.g.*: histone methylation, acetylation and ubiquitination, and G1 DNA damage checkpoint) and ATP metabolism processes such as purine ribonucleoside metabolic processes; b. Cytokinesis and different types of regulation of meiotic cell cycle regarding: sex determination, chromosome separation, telomere maintenance and polar body extrusion; c. Stem cell fate determination; d. Mitochondrial metabolism such as: cytochrome complex assembly and mithocondrial protein processing; e. Cell communication regarding: protein targeting and transport, cell-cell junction maintenance, membrane raft assembly, and immune system processes such as: T cell antigen processing and pattern recognition of the toll-like receptor 3; f. Brain development regarding: astrocyte, microglia, glial, myelin and synapse maturation; g. Skeletal muscle development; h. Protein modification *i.e*. methylation, acetylation,poly- and de-glutamylation); i. Several signaling cascades related to: cGMP catabolic process, epidermal growth factor-activated receptor activity, kinase A, TOR, GTPase, NIK/NF-kappaB, and keratinization; l. Metabolic processes especially chitin and hydrogen peroxide catabolic processes, ubiquitin dependent protein, bile regulation, thyroid hormon generation, and glycolytic process).

We used GOElite [33] to provide a biological description from the Gene Ontology for the 678 genes that were not found in the complete WikiPathways curated collection, and therefore not represented in the SNP-gene-pathway network. When we used all three main GO classes, molecular function, biological process and cellular component, 672 genes were annotated. For only 196 of these genes we found an annotation from the biological process tree and we focused on these. Although this resulted in less than half of the input genes being evaluated, this approach has the advantage that the annotation comes closest to a biological pathway description. The total number of biological processes identified for these 196 genes was 1,503. In order to further evaluate these annotated genes we also performed the same Gene Ontology annotation for the 368 genes from the SNP-gene-pathway network, and for these genes 4,544 biological processes GO terms were identified related to 351 of the 368 input genes. We compared these annotations of the two groups of genes. From the 1,503 annotations identified for the 362 genes not assigned by WikiPathways, 1,255 were observed previously for the 363 genes in the SNP-gene-pathway network. These 1,255 annotations are related to all the 351 genes with GO terms in the original SNP-gene-pathway network, and to the 122 out of 196 genes in the newly annotated group. This means that for the 74 (196–122) genes in the newly annotated group, there are biological process annotations not previously found. In total this added 248 (1503–1255) new biological process annotations to the T2DM SNP/gene set. A network visualization that illustrates the connections between these 74 genes and 248 GO terms is provided in Fig 3.

Two points related to the Gene Ontology comparison are worth noting: first, the 1,255 common terms extracted describe most of the original SNP-gene-pathway network and the corresponding 351 genes represent most of the pathways in that network. This implies that we have identified new information for the biological processes related to most of the pathways. Exceptions include the Aminoacid conjugation and Lnc-mRNA mediated mechanism of therapeutic resistance pathways for which we did not find any new related genes. To use the information about genes discovered by GWAS analysis for diabetes that are not in current pathways with similar Gene Ontology annotations as genes that are, and the information about discovered genes that have different gene ontology annotations, expert evaluation is required. This could lead to an extension of existing pathways if the discovered genes with similar annotations are in fact known to be involved in these pathways, or to development of new pathways that would cover processes found in the Gene Ontology not currently covered. In this way this information can be used to improve pathway curation, especially for pathways involved in the pathophysiology of T2DM. The second notable observation from the analysis of GO terms concern the connections between 248 biological processes exclusively linked to 74 genes that are not currently in any WikiPathways pathways are visualized for further analysis in Fig 3. Of course this network is likely to contain much of spurious information. If only one gene related to a biological process was found, this result is more likely to relate to a gene-process relationships not really related to T2DM. This could be because of: a false GWAS result, or the genes is involved in multiple processes or the SNP in that gene acts at a distance.

In order to have a general overview of the different biological processes in the network, related biological processes were clustered in the same frame of the Fig 3. Then, we constructed for each frame a GO ancestor chart of the biological terms, using the old version of QuickGO (http://www.ebi.ac.uk/QuickGO-Old/). In these charts GO slim terms were used to provide a summary of the results, which then gives an overview of the ontology content of the terms present in the tree. An example of the ancestor chart, with the GO slim terms related to the GO biological processes clustered in frame “l” of the Fig 3, is reported in S2 Fig.

In Fig 3 four frames functionalities referred to basic cell division and development such as: cytokinesis and different types of regulation of meiotic cell cycle regarding: sex determination, chromosome separation, telomere maintenance and polar body extrusion, stem cell fate determination, brain and skeletal muscle development. There is also a group of processes involved in cell communication regarding: protein targeting and transport, cell-cell junction maintenance, membrane raft assembly, and immune system processes such as: T cell antigen processing and pattern recognition of the toll-like receptor. Other groups are: DNA modification (*e.g.*: histone methylation, acetylation and ubiquitination, and G1 DNA damage checkpoint) and ATP metabolism processes such as purine ribonucleoside metabolic processes, mitochondrial metabolism such as cytochrome complex assembly, general functionalities regarding: protein modification (*i.e*. methylation, acetylation and poly- and de- glutamylation), regulation of metabolic processes related to: chitin and hydrogen peroxide catabolic processes, ubiquitin dependent protein, bile regulation, thyroid hormone generation, and glycolytic process. Finally, players of signaling cascade involved in cGMP catabolic process, epidermal growth factor-activated receptor activity, kinase A, TOR, GTPase, NIK/NF-kappaB, and keratinization. These results are an indication of relevant molecular processes in which the genes detected by the T2DM GWAS study play a role. Further literature investigation is required to expand the knowledge about the connection of these precesses and their genes in T2DM context, especially with respect to clinical measures of disease risk.

In conclusion, the Gene Ontology analysis performed for genes that currently were not assigned to pathways allowed us to identify 1) genes that are indeed related to several pathways identified by more fully annotated T2DM SNPs-genes, 2) genes that are functionally related in processes not covered currently in pathways, and 3) gene-process relationships that occur only occasionally (and are likely either to be of no real value to understand the disease development, or to have a conditional relationship with T2DM, such as via epistasis or gene-environment interactions).

### eQTL analysis

**Fig 4:**
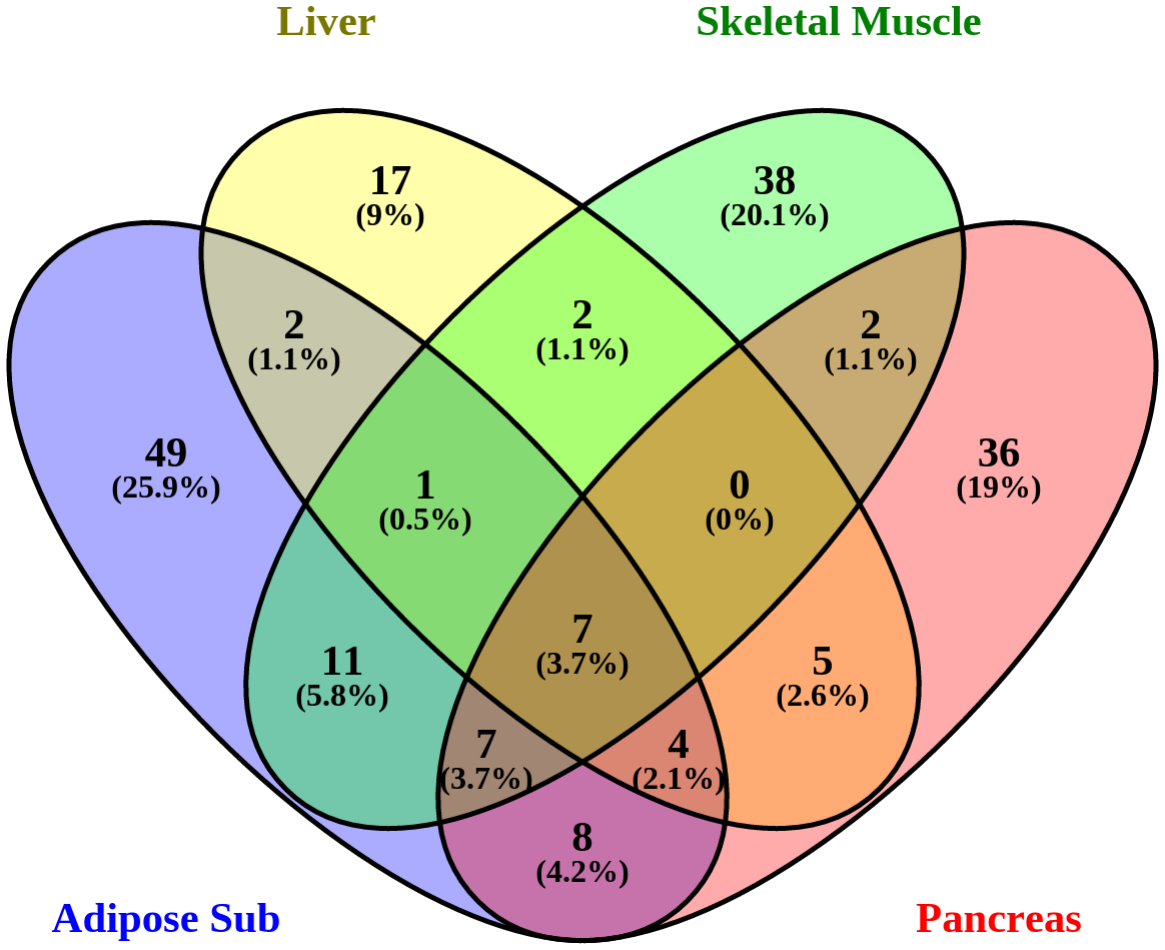
cis-eQTL in T2DM-relevant tissues. The Venn diagram indicates the numbers of cis-eQTLs in pancreas, liver, adipose subcutaneous and skeletal muscle, and the numbers shared among these tissues.

In order to evaluate which T2DM variants influence expression of the gene to which they map or any other genes, we used the GTEx portal to search for cis-eQTL SNPs within the T2DM SNP set. We first checked if any of the 716 SNPs that did not map to a specific gene had GTEx data indicating an eQTL function. We then evaluated whether the SNPs that influence expression of a gene are known in GTEx to affect cis-eQTLs in subcutaneous adipose tissue, liver, pancreas or skeletal muscle. For each tissue we selected the genes from cis-eQTLs with a p-value minor and egual of 0.05 (S6 Table), and we visualized the results in a Venn diagram in Fig 4. The Venn diagram shows that most of the cis-eQTLs are tissue-specific: 36% in pancreas, 26% in adipose subcutaneous, 20% in skeletal muscle and 9% in liver. This result confirms previous findings suggesting that GWAS variants are enriched related to tissue-specific cis-eQTLs [15]. The selected tissues are relevant in T2DM, and we checked the detected genes, finding well-known T2DM genes such as: *PPARG* and *TCF7L2* in pancreas or *APOB* and *LPL* in subcutaneous adipose tissue.

Moreover, we found 45 cis-eQTLs genes shared between at least two tissues. Within this set of genes we found only one gene (*MAP3K8*) present in the core of the network of Fig 2 because it has a high number of pathway connections, and it is present in several important pathways, such as: Insulin signaling (WP481) leading to a cell growth differentiation, and TNF-alpha signaling (WP231) ending to the activation of *NFKB*. A significant positive effect of diabetes related SNPs was found for *MAP3K8* expression in pancreas and liver, in adipose tissue there was a tendency towards a negative effect on expression but the p-value was only 0.45. The *MAP3K8* gene encodes a kinase that in adipocytes is involved in inflammatory cytokine–induced ERK1/2 activation, and deregulation of its expression suggests a role in adipose tissue dysfunction in obesity [48]. However, *MAP3K8* does not activate insulin in adipose tissue and does not trigger its effects like lipolysis. The presence of *MAP3K8* in a diabetes related eQTL in liver and pancreas could still lead to a significant alteration of the downstream insulin signaling pathway. For instance in the Angiopoietin Like Protein 8 Regulatory Pathway (WP3915), *MAP3K8* is activated via the insulin cascade together with other MEK/MAP kinases and another downstream effect, beside cell differentiation, is the activation of the *ANGPTL8* gene that is known to be a relevant regulator of glucose and lipid metabolism in liver, and it is identified as a novel drug target for treatment of T2DM and dyslipidemia [49].

Finally, we checked the common genes identified in the three approaches: the cis-eQTL tissue-specific lists, the gene-environment analysis and the highly connected pathway based network. We identified three genes and their (partner) SNPs present in all three lists: *PPARG* in pancreas cis-eQTL (rs1801282), and *LPL* (rs328) and *APOB* (rs693) in adipose tissue cis-eQTL. *PPARG* is a transcription factor and the molecular target of the insulin-sensitizing drug, thiazolidinedione; its variant rs1801282 is robustly associated to reduced risk of T2DM in different populations [50]. In contrast, *LPL* and *APOB* variants are not (yet) considered to be associated with T2DM [4], but in the CardioGxE database they show association with diabetes related traits such as: *LDL* cholesterol and triglycerides level for *APOB* and HDL-cholesterol and triglycerides level for *LPL*. In particular, the *LPL* rs328 SNP was previously mentioned to have a high impact on sequence consequences according to VEP, because it is a nonsense mutation that truncates the *LPL* protein to 446 instead of 448 amino acid residues. Despite the high impact effect assigned to this *LPL* nonsense mutation, there are controversial results from *in vitro* studies that show either a normal enzyme activity of the *LPL* protein with the missing codon at the carboxyl-terminal [41] or that the mutation might be responsible for a defect in lipid interface recognition [42]. Yet another study examined the association of *LPL* with T2DM in a Korean population taking into account different *LPL* SNPs including rs328, that had significant associations with blood glucose-related phenotype [51]. In conclusion, our finding still supports the relevance of this and other genes like *APOB* in the context of T2DM.

### Conclusions

In this Bioinformatics study we were able to re-analyze the output of previously published Type 2 Diabete Mellitus (T2DM) GWAS studies by integrating several types of biological information regarding the GWAS SNPs and the genes in which the variants are located, with the relevant findings summarized in Table 1. We also took advantage of network visualization to recognize different types of biological relationships, such as: genes highly connected with pathways, and overlapping genes that share the same SNP GWAS hit.

**Table 1:** Summary of the relevant genes detected in the secondary analysis of T2DM GWAS study.

Type of gene detection	Gene name
Genes with nonsense SNP	LPL, MMP26
Genes with missense SNPs	MMP26, KCNJ11, VSTM4, ART5, TBX15
Genes with synonimous SNPs	OR51A7, SVIL, ARF3, PLEKHG7, VSIG10, RP11-302B13.5
Genes highly connected with pathways	27 genes listed in S1 Table
Genes disconnected from the SNP-gene-pathway central network in Fig 2	VMP, TSEN, SYT, SET, RPP3, RIMS, NR3C, NFI, NDUFS, MPC, MBD, HSD17B, FADS2, CPLX2
Genes overlapping the same SNPs	41 genes listed in S3 Table
Gene highly detected by significant GWAS SNPs	CDKAL1
Genes with significant Gene-Environment interaction	35 genes listed in S4 Table
Genes detected by GTEX cis-eQTLs in adipose, liver, pancreas, and skeletal muscle tissue	264 genes listed in S5 Table
Genes with common GTEX cis-eQTLs, Gene-Environment interaction and high pathway connection	PPARG, LPL, APOB

First, we identified pathways relevant in T2DM. We then analyzed a SNP-gene-pathway network that provided information about the types of biological processes in which the GWAS genes have a role. We combined this with other information about the biological roles of the genes in the network obtained from various sources (CardioGxE, GTEx, DiSGeNET). We propose these network steps as a method complementary to the standard pathway analysis, because it allows the visualization of the relationships between GWAS genes, different functional annotations, the relevant SNPs, and pathways containing these. Next, we selected a number of relevant genes that are featured in small SNP-gene network nodelets (basically the edges in these nodelets consist of SNPs known to be located in more than one gene). This highlights the SNPs that can affect multiples genes. Sometimes choices for one or the other interaction can be made based on the observed phenotype and the function of the affected genes.

Furthermore, we addressed the T2DM GWAS genes that were not included in the WikiPathways collection, and as a consequence were not analyzed in the SNP-gene-pathway network. For those genes the annotation tree of biological processes GO terms were analyzed, and a list of relevant terms linked to 74 T2DM GWAS genes is provided as a network visualization for further investigation. Next we created a connection map based on the GO biological process annotation shared by both the 351 genes found in pathways and the 122 genes not found in pathways. Apart from their direct utility to study the relationship between disease related genes and their annotated functions in GO, such connectivity maps are also useful to evaluate which disease relevant processes have not been captured well in pathway collections.

Finally, we used three more disease targeted knowledge resources to select relevant genes in the SNP-gene-pathway network that are known to share more specific biological functionality. 1) The CardioGxE database depicted genes involved in specific environmental interactions, but also associated with T2DM related traits. 2) eQTL analysis provided an idea of the influence that the variants can have on the expression of the genes in the network at the tissue level. 3) DisGeNET shows the evidence regarding the gene-disease association and applying this suggested to us those genes in the SNP-gene-pathway network that do not have compelling evidence to confirm a T2DM role.

A combination of the approaches described allowed us to identify genes such as *LPL* and *APOB*, of which the variants appear to play an important role in T2DM but have not been well studied in this context so far.

## Supporting information

S1 Fig SNP-gene network. The image visualizes 39 SNPs (green triangles) located in 41 genes (pink circles). The SNP-gene relation is represented by 19 gene-SNP-gene structures in which multiple genes overlap with the same SNPs.
(TIF)

S2 Fig Ancestor chart of GO biological processes. The tree represents the relations of the GO biological processes identified in the frame “l” of the Fig 3. The colorful boxes are the GO slims terms: a cut-down version of the GO ontologies containing a subset of the terms in the whole GO chart.
(TIF)

S1 Table T2DM SNPs from the GWAS Catalog. The 757 T2DM SNPs retrieved from the GWAS Catalog reported with genes and pathways found using the workflow described in this study.
(XLSX)

S2 Table Highly connected genes. The table lists 27 genes with a pathway connection higher than ten, and if the gene is positioned in the core of the main SNP-gene-pathway network. Evidence of gene-disease association and relative strength score from DisGeNET database are reported.
(XLSX)

S3 Table Overview of the SNP-gene connection. (XLSX)

S4 Table Genes-SNP-gene structure. The table shows genes that overlap with other genes and they have in common one or more T2DM GWAS SNPs. Evidence is reported for the DisGeNET score and PubMed ID of the gene involved in T2DM.
(XLSX)

S5 Table Significant gene-environment interactions, as mined from CardioGxE. (XLSX)

S6 Table Significant genes detected in the cis-eQTLs database with positive effect size in adipose, liver, pancreas and skeletal muscle tissue. (XLSX)

S7 Table Meta-analysis on Genetic Association Studies Checklist from PLOS ONE. (XLSX)
