# Supplementary figures and images for: From SNPs to pathways: Biological interpretation of type 2 diabetes (T2DM) genome wide association study (GWAS) results
Source: PLoS One. 2018 Apr 4;13(4):e0193515. doi: 10.1371/journal.pone.0193515 (PMC5884486; doi:10.1371/journal.pone.0193515)

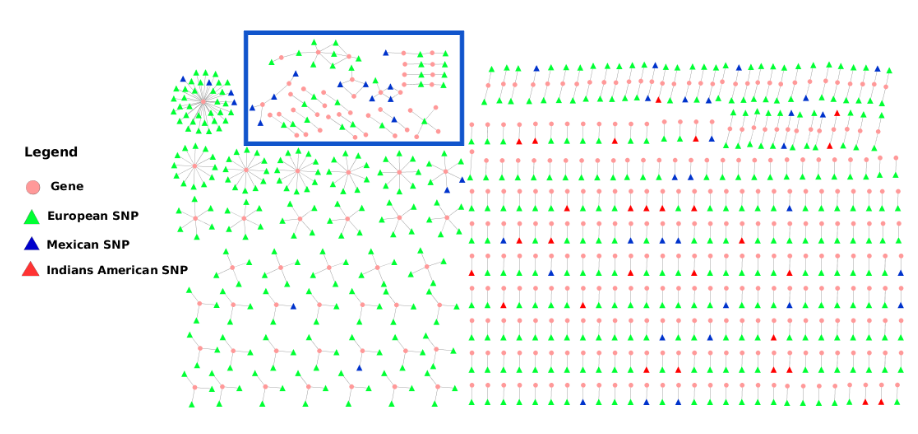

Supplement: S1 Fig — The image visualizes 39 SNPs (green triangles) located in 41 genes (pink circles). The SNP-gene relation is represented by 19 gene-SNP-gene structures in which multiple genes overlap with the same SNPs. (TIF) [file pone.0193515.s001.TIF]

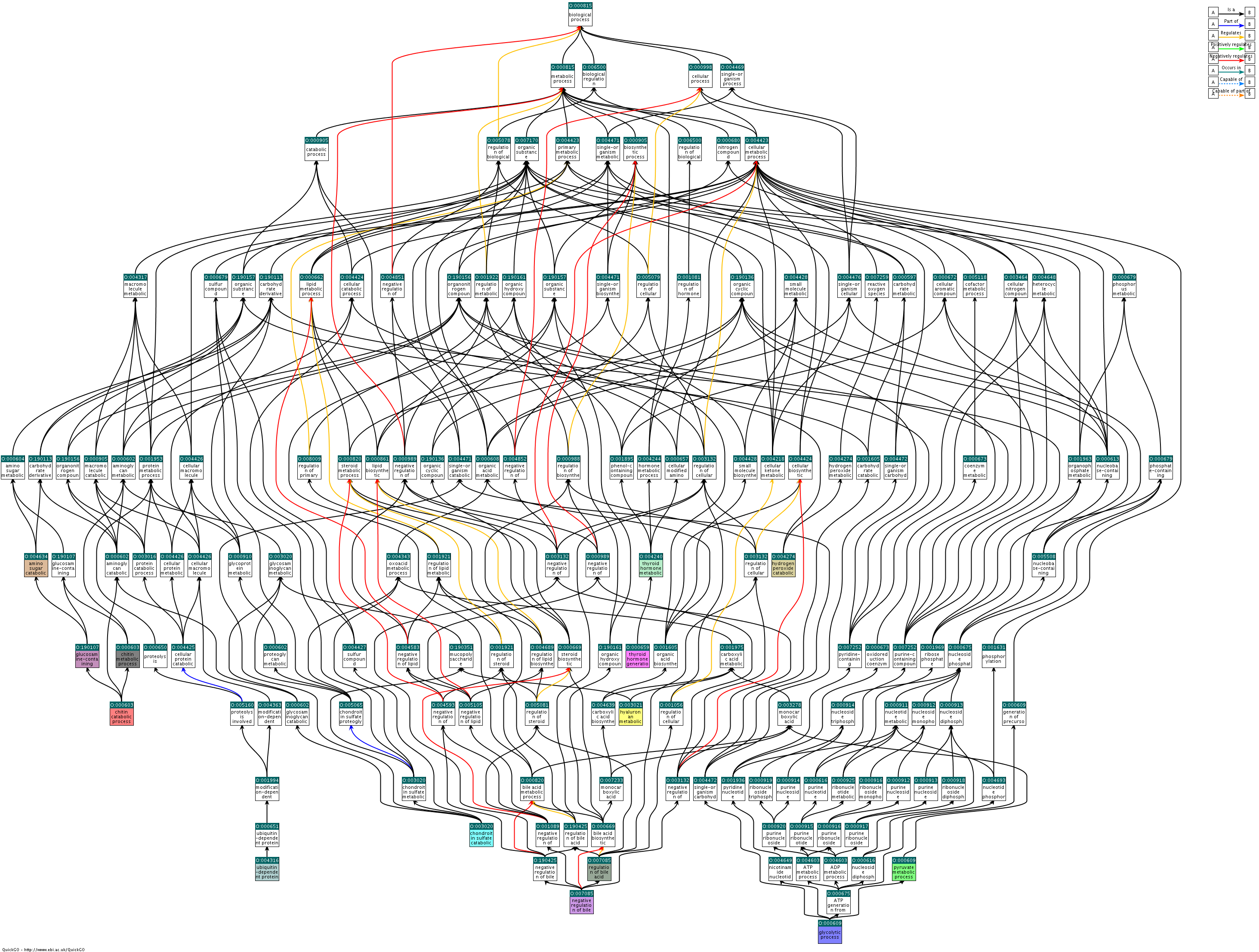

Supplement: S2 Fig — The tree represents the relations of the GO biological processes identified in the frame “l” of the Fig 3. The colorful boxes are the GO slims terms: a cut-down version of the GO ontologies containing a subset of the terms in the whole GO chart. (TIF) [file pone.0193515.s002.tif]
